# Genetic deletion of α7 nAChRs reduces hippocampal granule and pyramidal cell number in both sexes but impairs pattern separation in males only

**DOI:** 10.3389/fnins.2023.1244118

**Published:** 2023-09-07

**Authors:** Ayland C. Letsinger, Samir A. Nacer, Korey D. Stevanovic, Gary J. Larson, Jemma S. DeFilipp, Jesse D. Cushman, Jerrel L. Yakel

**Affiliations:** ^1^Neurobiology Laboratory, National Institute of Environmental Health Sciences, National Institutes of Health, Durham, NC, United States; ^2^Social & Scientific Systems, Inc., a DLH Holdings Corp. Company, Durham, NC, United States

**Keywords:** CHRNA7, acetylcholine, stereology, cognitive flexibility, touchscreen

## Abstract

**Introduction:**

Neurogenesis within the dentate gyrus is thought to play an important role in cognitive processes such as reversal learning and pattern separation. The α7 nicotinic acetylcholine receptor (α7 nAChR) is expressed early in newly formed granule cells of the dentate gyrus, though its role in neurogenesis and related cognitive function is not fully understood.

**Methods:**

To better characterize relevant function of α7 nAChRs, we performed unbiased stereology to quantify hippocampal granule cells, pyramidal cells, and total volume and used a touchscreen operant spatial discrimination/reversal task to test pattern separation in a global α7 nAChR knockout mouse line.

**Results:**

The knockout resulted in an ≈22% reduction in granule cells and a ≈ 20% reduction in pyramidal cells in both sexes, with no change in total hippocampal volume. However, the knockout impaired performance in the touchscreen task for males only. The sex-dependent difference in behavioral, but not stereological, results suggest a divergence in the structure–function relationship in males versus females. Detailed analyses revealed males were more biased by the initial reversal contingency relative to females indicating a potential source of the sex-specific interaction with the loss of α7 nAChRs.

**Discussion:**

These findings argue that the α7 nAChR plays a critical role in hippocampal development, not just granule cell neurogenesis, and plays a sex-dependent role in cognitive function.

## Introduction

Dysfunctional memory encoding associated with dementia has been hypothesized to involve impairment of adult neurogenesis within the dentate gyrus (Briley et al., 2016; Moreno-Jiménez et al., 2019). Lesions of hippocampal granule cells in rodents lead to deficits in spatial pattern separation (McTighe et al., 2009; Talpos et al., 2010; Delotterie et al., 2015). Moreover, preventing adult neurogenesis while maintaining existing granule cells impairs memory performance in rodents, particularly in tasks that require high level of spatial and/or contextual discrimination (Clelland et al., 2009; Nakashiba et al., 2012; Zhuo et al., 2016). Thus, understanding what mechanisms are responsible for dysfunctional neurogenesis, specifically the proliferation and maintenance of granule cells, will help provide therapeutic targets and guide development of interventions to protect individuals from memory loss.

One mechanism for impaired neurogenesis within the dentate gyrus lies in the diminished cholinergic tone found in the majority of individuals suffering from dementia (Davies and Maloney, 1976; Schliebs and Arendt, 2011; Fontana et al., 2023). In rodents, the inhibition of local cholinergic activity via acetylcholinesterase (Chacón et al., 2003) or halorhodopsin (Zhu et al., 2017) reduces the survival of granule cells and impairs memory performance. Thus, acetylcholinesterase inhibitors, which decrease the breakdown of acetylcholine, are currently the most commonly prescribed drugs to alleviate dementia symptoms, although the benefits are modest and side effects such as nausea and gastrointestinal discomfort are common (Mohammad et al., 2017). Acute nicotine exposure can also alter and even enhance memory function in humans and rodents (Newhouse, 2004). A mutual target of acetylcholine and nicotine are the nicotinic acetylcholine receptors (nAChRs). The function of nAChRs in the brain is highly complex as they are expressed in most neuron populations including granule cells, pyramidal cells, and GABAergic interneurons, as well as non-neuronal cells including astrocytes, microglia, macrophages, and endothelial cells (Letsinger et al., 2022).

The α7 nAChR subunit has been shown to support neurogenesis in multiple ways such as altering plasticity (Gu and Yakel, 2011; Gu et al., 2012), regulating GABA production through PKA phosphorylation (Bates et al., 2014), reducing inflammation (Conejero-Goldberg et al., 2008), and increasing levels of IGF-1 (Kita et al., 2014). A genetic knockout of α7 nAChRs in mice led to granule cells with truncated dendritic arborization and immature GABAergic currents (Campbell et al., 2010). Additionally, work from our lab has demonstrated that knockout of α7 nAChRs in nestin-positive neural stem cells results in fewer neural stem cells within the subgranular zone of the dentate gyrus, a slight overall increase in neurogenesis markers, and impaired performance in a radial arm maze task in male mice, but not females (Otto and Yakel, 2019). In addition, we showed that the knockout of the α7 receptor in GAD2-expressing cells (GABAergic interneurons) decreases the presence of radial glial-like cells in male mice only, but impaired spatial memory in both sexes (Nacer et al., 2021).

Despite prior focus on the role of α7 nAChRs in granule cell neurogenesis, the extent to which α7 nAChR function impacts the total number of hippocampal cells and overall hippocampal volume has yet to be quantified. We therefore sought to characterize the role of α7 nAChRs on hippocampus structure formation, by performing unbiased stereology to quantify hippocampal granule cells, pyramidal cells, and total volume in a global α7 nAChR knockout mouse line. In addition, we characterized the behavioral performance of global α7 nAChR knockout mice in a translational pattern separation operant touchscreen task that is known to be dependent on hippocampal neurogenesis (Clelland et al., 2009). We hypothesized that a global knockout of α7 nAChRs would lead to diminished granule cell numbers specifically in male mice, thereby causing performance deficits in the challenging small separation phase of this task.

## Methods

### Animals

**Figure 1:**
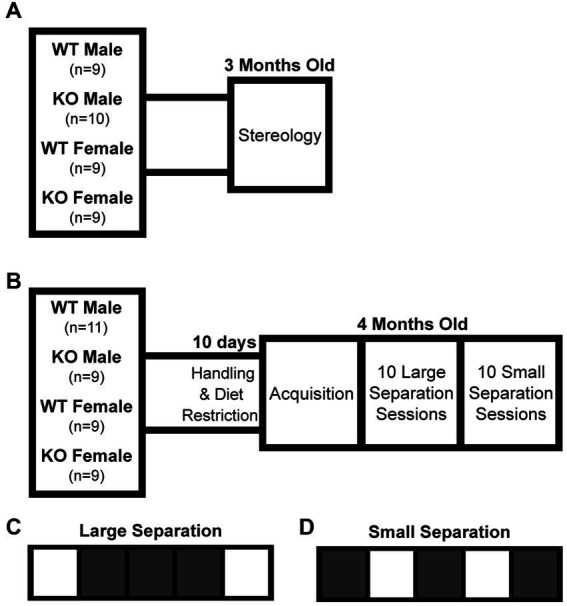
Experimental timeline and touchscreen task paradigm. **(A)** The first cohort of mice were used for stereological analysis of total hippocampal granule cell count, pyramidal cell count, and volume at 3 months old. **(B)** The second cohort of mice were tested for pattern separation function at 4 months old. **(C)** The large separation task considered to not be dependent on dentate gyrus function. **(D)** The small separation task considered to be dependent on dentate gyrus function. WT, wildtype; KO, global α7 nAChR knockout.

B6.129S7-Chrna7^tm1Bay^/J^1^ male and C57Bl/6 J^2^ female mice were purchased (The Jackson Laboratory, Bar Harbor, ME) and bred in house to produce heterozygous α7 nAChR offspring. These offspring were bred together to produce homozygous knockouts (KO), heterozygous partial knockouts (not used in this study), and wildtype (WT) offspring. The B6.129S7-Chrna7^tm1Bay^/J strain is a result of deleting exons 8–10 of the *Chrna7* gene (Orr-Urtreger et al., 1997). Genotypes were determined and validated from tail clip biopsies obtained at weaning (Transnetyx, Cordova, TN, United States). All animals were housed in same-sex sibling cohorts of at least three per cage. The first cohort of mice generated for stereology at 3 months old, an age where brain growth has reached a plateau, were allowed food and water *ad libitum* and were not handled or exposed to any behavioral task (Figure 1A). A second cohort of mice began diet restriction (85–90% initial mass) and daily handling 10 days prior to behavioral testing at 4 months old (Figure 1B). This age was chosen based on scheduling and being a comparable age to the stereology cohort. All procedures were approved and performed in compliance with the NIEHS/ NIH Humane Care and Use of Animals Protocols.

### Tissue preparation and unbiased stereology

At 3 months old, the first cohort of mice were deeply anesthetized using sodium pentobarbital (FatalPlus; 100 mg/kg) and transcardially perfused using ice cold 0.1 M phosphate buffer saline (PBS; pH 7.4 with 0.1% heparin) followed by ice cold 4% paraformaldehyde (PFA) in PBS. The entire skull was left in 4% PFA for 48 h after perfusion to prevent the likelihood of dark neuron artifacts (Jortner, 2006). Dissected brains were then cryoprotected at 4°C in a 30% sucrose and 0.1 M PBS solution. Brains were then transferred to Charles River Laboratories, Inc. (Durham, NC, United States).

Trimmed brains were embedded in paraffin in a coronal/transverse orientation. After a random start, two 3 μm thick disector pairs, a section for hematoxylin and eosin staining, and a reserve section were collected at 270 μm intervals, which produced four slides per interval, with approximately 8–12 intervals per mouse. The disector pair from each interval was stained with Cresyl Violet. Stained disector pairs were scanned at 40X magnification using a Nanozoomer whole slide scanner (Hamamatsu Photonics K.K., JP) and imported into the Autodisector platform (Visiopharm, DK).

**Figure 2:**
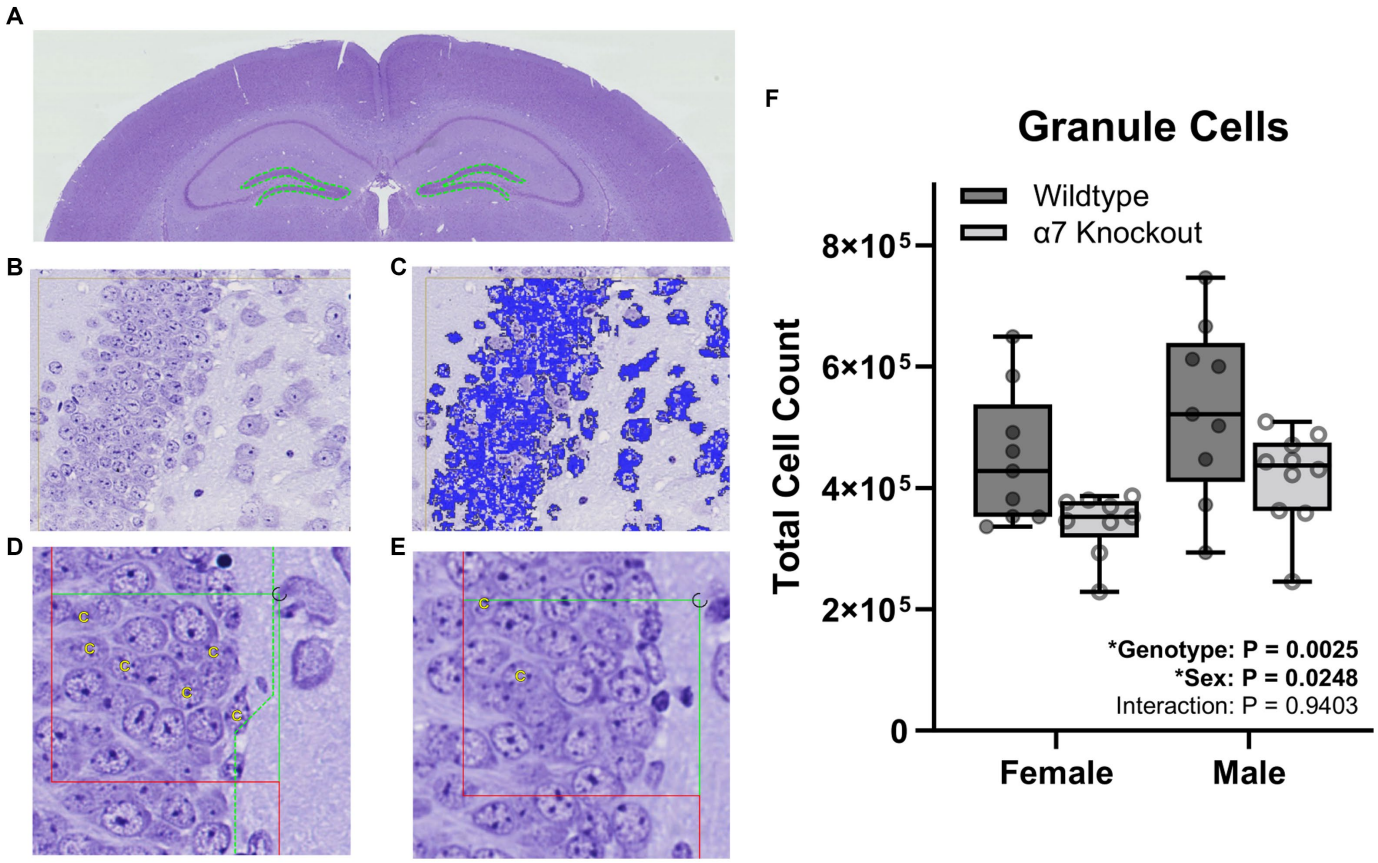
Stereological granule cell count within the dentate gyrus. **(A)** A ROI (dashed green line) was manually generated around the dentate gyrus of the hippocampus. **(B)** Proportionator sampling was performed within the dentate gyrus at 40X magnification. **(C)** Granule cells (blue) were manually selected with the help of an analysis algorithm within the ROI. **(D,E)** Granule cells were counted and marked with a “C” when present in either the reference section **(D)** or the look-up section **(E)**, but not present in both sections. **(F)** Granule cell counts. Boxplots represent median, interquartile range, absolute range, and individual points for each animal. ^*^represents a significant effect at *p* < 0.05 for that effect.

**Figure 3:**
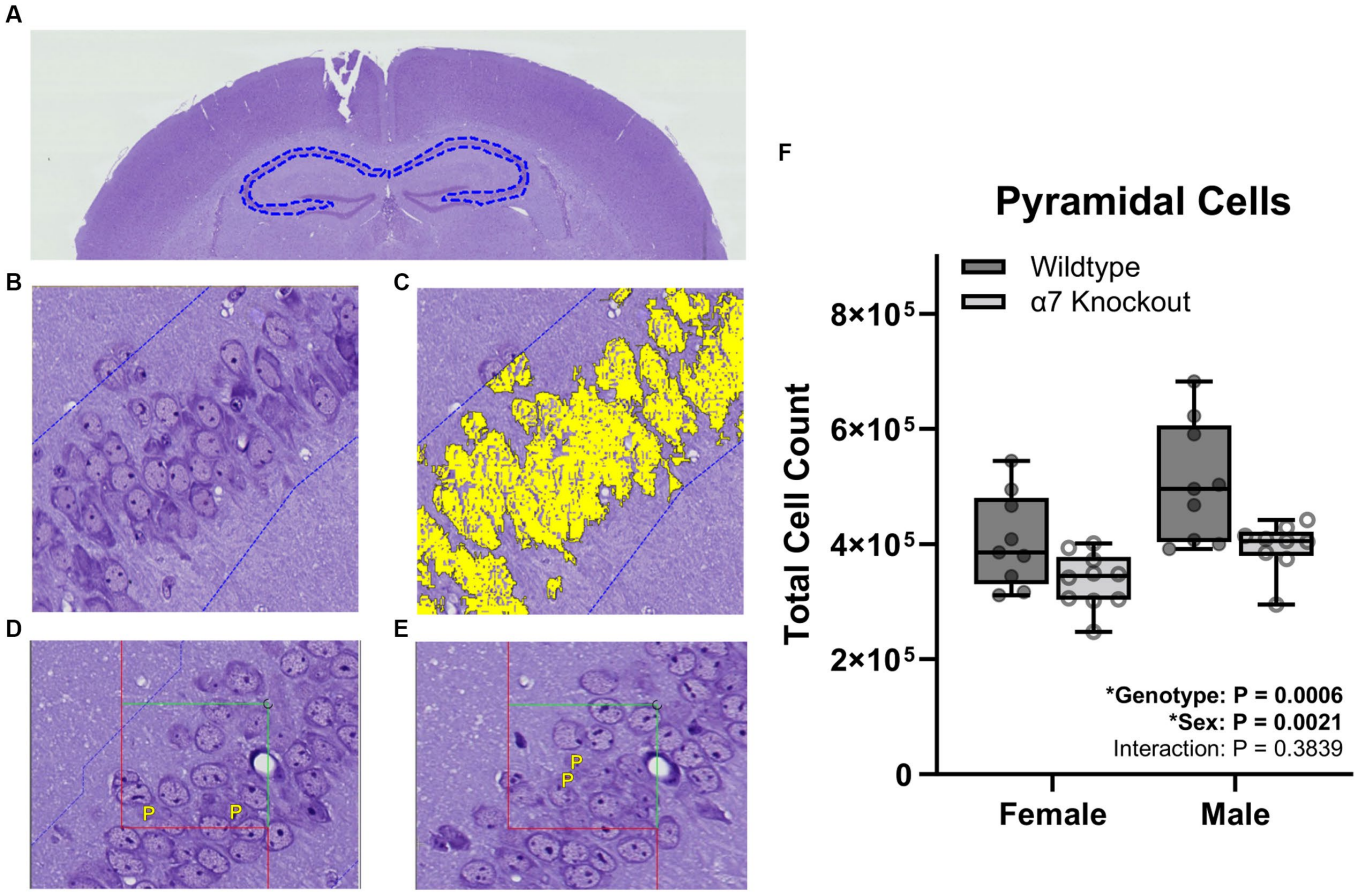
Stereological pyramidal cell count within the CA1-3. **(A)** A ROI (dashed blue line) was manually generated around the CA1-3 of the hippocampus. **(B)** Proportionator sampling was performed within the CA1-3 at 40X magnification. **(C)** Pyramidal cells (yellow) were manually selected with the help of an analysis algorithm within the ROI. **(D,E)** Pyramidal cells were counted and marked with a “P” when present in either the reference section **(D)** or the look-up section **(E)**, but not present in both sections. **(F)** Pyramidal cell counts. Boxplots represent median, interquartile range, absolute range, and individual points for each animal. ^*^represents a significant effect at *p* < 0.05 for that effect.

For granule cell counts, sections were aligned and regions of interest (ROI) were manually drawn around the dentate gyrus (Figure 2A). For pyramidal cell counting, sections were aligned and ROI were manually drawn around the three fields of the CA1-3 (Figure 3A). For each cell type, three independent samplings were performed using seven fields randomly selected from the 8–12 intervals with an unbiased counting frame of 3,000 μm^2^ (60 μm × 50 μm). Sampled fields were loaded into the Stereology module of the Visiopharm software and the number of granule or pyramidal cells were manually counted with the help of an analysis algorithm for each animal. Cell types were clearly identified by a 5–20 μm diameter elliptical soma with a granular nucleus. Individual nuclei were identified within the unbiased counting frame by the presence of a nuclear membrane and an unobstructed view of a portion of the interior of the nucleus (Figures 2B,C, 3B,C). Cells were counted in both directions, i.e., if the nucleus in a stained cell was present in either the reference or the look-up section and not the other, that cell was counted (Figures 2D,E, 3D,E).

The total counts across all sampling fields for granule and pyramidal cells were summed by the software separately. The weighted counts were calculated by the software by multiplying the raw counts in each sampling field by the inverse of the probability of having a positive count in that field (Gardi et al., 2008). Data was imported into the Visiopharm software calculator. For each independent sampling, the total number of granule or pyramidal cells were calculated according to the equation N = ∑Q/2 × 1/ssf; where ∑Q was the sum of the weighted counts (divided by 2 as counting was performed in both directions of the dissector), and ssf was the section sampling fraction, which is the section thickness divided by the sampling interval (Gardi et al., 2008). The mean and standard deviation of the three samplings was calculated for each animal. The coefficient of error (CE) of the three independent samplings was also calculated for each animal according to the equation CE = STD/(mean × √2) (Gardi et al., 2008). To determine whether the precision of the estimate was adequate, the precision range of an optimally balanced estimator (PROBE) calculation was performed for each estimate (Løkkegaard, 2004). The average CE and coefficient of variation (CV) for each group were determined for each estimate and the following equation was performed: PROBE = CV2/CE2. A PROBE value of greater than 2 indicates that the biological variability, represented by the coefficient of variation, is sufficiently larger than the sampling error, represented by the coefficient of error, that any difference may confidently be attributed to the variability between animals. All estimates were below the *a priori* PROBE value of 2.

**Figure 4:**
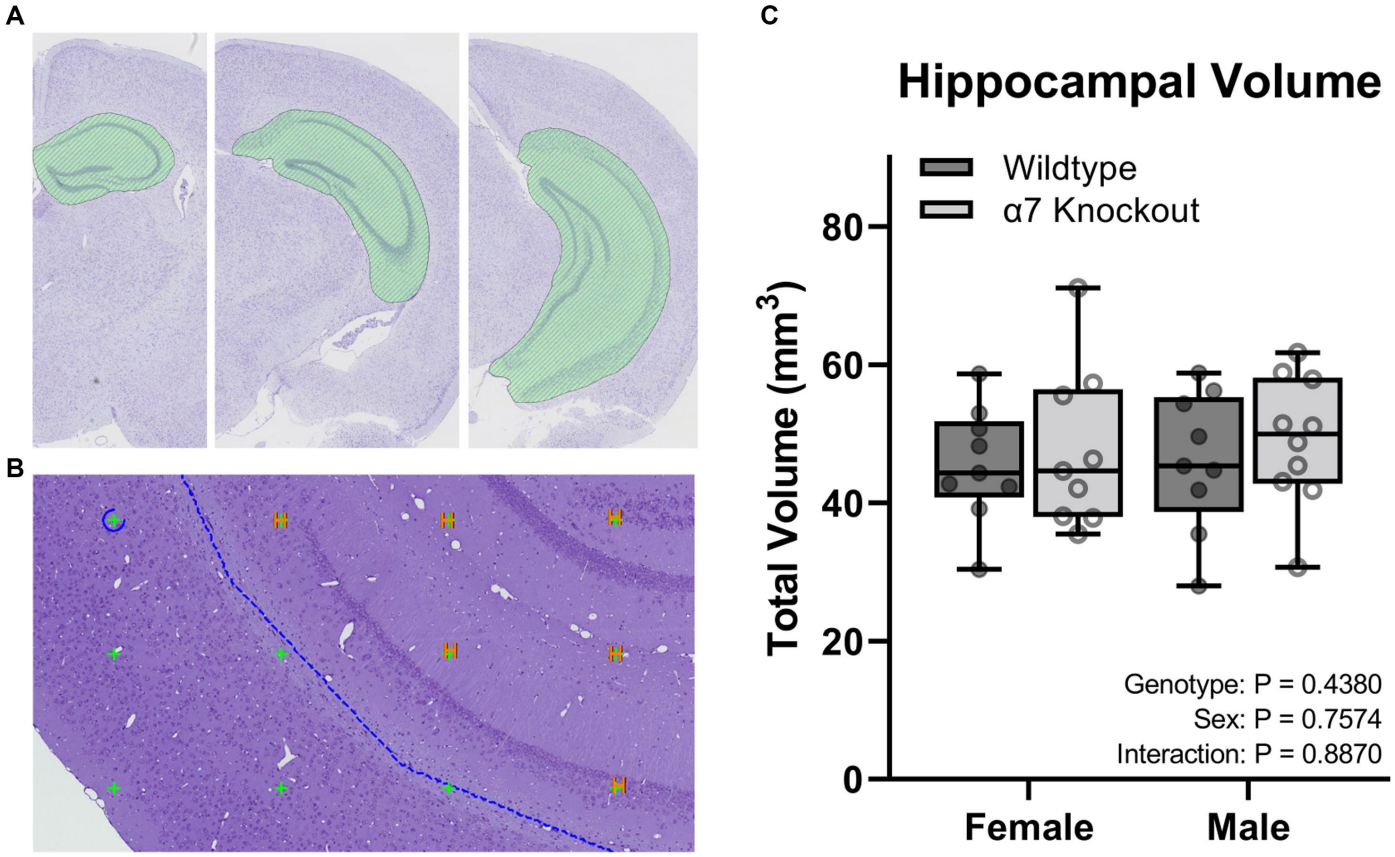
Stereological hippocampal volume. **(A)** A ROI (green curvilinear shape) was manually generated around the CA1-3, dentate gyrus, and the subiculum of the hippocampus. **(B)** Points touching the hippocampus were tagged with an “H” and used to calculate the total volume. **(C)** Volume of the hippocampus. Boxplots represent median, interquartile range, absolute range, and individual points for each animal.

To calculate hippocampal volume, sections were aligned, and ROI were manually drawn around the CA1, CA2, CA3, dentate gyrus, and the subiculum of each section (Figures 4A,B). Three-dimensional tissue shrinkage during processing was estimated by the following formula: 3D global shrinkage = 1 −(post-processing weight/pre-processing weight) (Gundersen et al., 2013). The number of points hitting the hippocampus (tagged H) were summed by the software and imported into the calculator module for total volume estimation (Figure 4B). The volume of each sampling was calculated according to the Cavalieri method using the following equation: V = ΣP × A(p) × T; where ΣP was the number of intersecting points, A(p) was the area per point (0.269992 mm^2^), and T was the sectioning interval (Howard and Reed, 2005). The volume for each animal was corrected for shrinkage using the equation V_corrected_ = V_estimated_/(1–3D shrinkage) (Gundersen et al., 2013). Noise was calculated as: Noise = 0.0724 * (*b*/√*a*) * √(*n* * ΣP); where *n* is the number of sections and *b*/√*a* is the average profile shape, where *b* is the perimeter, and *a* is the profile area. The profile shape was determined to be 4. Noise was combined with the estimator variance of Σ_area_ to produce a total variance, which was used to calculate a coefficient of error (CE) for each estimate: CE = √(total variance)/ΣP (Gundersen and Jensen, 1987).

### Touchscreen assay

Bussey-Saksida touchscreen operant chambers (Loughborough Campden Instruments, United Kingdom) housed within sound attenuating cubicles (MED-OFA-017, Med Associates Inc., Fairfax, VT, United States) were utilized to test pattern separation. This touchscreen task was generated to mimic the Cambridge Neuropsychological Test Automated Battery (CANTAB), a highly selective test for dementia in humans (Sahgal et al., 1991; Égerházi et al., 2007) in which the ability to discriminate choices has been found to be dependent on hippocampal neurogenesis (Clelland et al., 2009). Both tests require the subject to touch visual cues in a pattern to precisely test spatial memory combined with immediate reward. A 5-Choice Mask (4 × 4 cm each with 1 cm spacing) was utilized for both initial training and position discrimination tasks. The operant chambers (25 cm × 20 cm × 14 cm) contained a touch screen (25 cm × 15 cm) on one wall and a reward-port on the opposing wall. The reward stimulus (evaporated milk) was presented using a peristaltic pump. Training began with an “Initial Touch” phase where one of the five squares was illuminated (at random) for up to 10 s. A 21 μL reward was presented if the mouse touched the illuminated square during the 10 s. If no touch occurred, the illuminated square was turned off and a 7 μL reward pulse was presented. The next phase of training was a “Must Touch” similar to the previous phase except no reward was presented in the event the illuminated square was not touched. All mice were trained to a criterion of performance requiring greater than 85% correct performance over 2 consecutive days before moving to the next phase. After successful completion of the training, the mice were then tested for 10 days on the Large Separation task (Figure 1C). In this task, either the far right or far left illuminated squares, separated by three dark squares, were designated as correct. The contingency reversed after seven correct touches in eight attempts. The mice were then given 10 days of the Small Separation task (Figure 1D) where either the left or right illuminated squares, separated by a single dark square, were designated as correct. Like the Large Separation task, the contingency reversed after seven correct touches in eight attempts. For both tasks, the next trial started 2 s after reward was consumed (when the correct poke was made) and 10 s timeout was imposed when an incorrect poke was made (Supplementary Video 1).

### Statistics

For stereology data, two-way ANOVAs with genotype and sex as factors were employed to determine differences in hippocampal cell counts and volume with α set at 0.05. For the touchscreen data, repeated measures ANOVAs with genotype and sex as the primary factors to determine differences in the average percentage of correct attempts were employed, with α set at 0.05. Tukey *post hoc* tests were used in the case of significant effects. After initial data analysis, additional analyses were performed to determine differences in motivational variables: reward latency and selection latency with α set at 0.05. To characterize behavioral strategies, a generalized linear model (GLM) logit link function (logistic regression) adapted from Chen et al. (2021) was used to predict left vs. right choice based on which side was chosen previously and whether it was rewarded based on the following equation (Chen et al., 2021). Genotypes were blinded to experimenters throughout the experimental phase but not during analyses.


$$\log\frac{P\left(L_{t}\right)}{1-P\left(L_{t}\right)}=\beta_{0}+\beta_{1}\;\cdot \;\;\left(L_{t-1}-R_{t-1}\right)+\beta_{2}\cdot O_{t-1}\;\cdot \;\;\left(L_{t-1}-R_{t-1}\right)$$

$$\begin{array}{l} L_{t},R_{t},\;\;and\;O_{t}\;are\;binaryvariablesequalto\;0\;or\;1,\\ indicatingthesidechoiceandoutcomeoftrial\;t: \end{array}$$

$L_{t-1}=1$ and $R_{t-1}=0$ if the left side was chosen at the previous trial.

$L_{t-1}=0$ and $R_{t-1}=1$ if the right side was chosen at the previous trial.

$O_{t-1}=1$ if the previous trial’s side choice was correct, otherwise $O_{t-1}=0$ .

β_0_ to represent an overall side bias (left versus right).

β_1_ to represent the tendency to stay or switch sides regardless of the previous reward outcome.

β_2_ to represent the tendency to stay or switch sides based on the previous reward outcome.

Trials were concatenated across sessions and a single GLM was fit to each animal’s cumulative set of trials. The final beta coefficient estimates values incorporating all trials were analyzed utilizing two-way ANOVA to assess overall strategy differences as a function of sex and genotype. R (Version 4.2.2) was used to fit the models. GraphPad Prism (Version 8.4.1, Graphpad Software Inc., La Jolla, CA, United States) was used to perform remaining statistics and generate figures. Individual data and statistics are displayed in Supplementary Table 1.

## Results

### A global α7 nAChR KO decreases hippocampal granule and pyramidal cell counts but not total volume

To determine if hippocampal structure is affected by the absence of α7 nAChRs, we performed unbiased stereology to quantify hippocampal granule cells (Figure 2), pyramidal cells (Figure 3), and total hippocampal volume (Figure 4) in female and male α7 nAChR KO and WT mice. For granule cells (Figure 2F), females had on average 17% less total cells than males [*F*(1, 33) = 5.534, *p* = 0.0248], KOs had on average 22% less than WTs [*F*(1, 33) = 10.76, *p* = 0.0025], and an interaction was not significant [*F*(1, 33) = 0.005702, *p* = 0.9403]. For pyramidal cells (Figure 3F), females had on average 18% less total cells than males [*F*(1, 33) = 11.12, *p* = 0.0021], KOs had on average 20% less than WTs [*F*(1, 33) = 14.24, *p* = 0.0006], and an interaction was not significant [*F*(1, 33) = 0.7789, *p* = 0.3839]. For hippocampal volume (Figure 4C), there was no significant effect of sex [*F*(1, 33) = 0.09706, *p* = 0.7574], genotype [*F*(1, 33) = 0.6165, *p* = 0.4380], or an interaction [*F*(1, 33) = 0.02050, *p* = 0.8870]. For body mass, females were 23% lighter than males [*F*(1, 34) = 62.30, *p* < 0.0001] and there was no effect of genotype [*F*(1, 34) = 1.408, *p* = 0.2436] or an interaction [*F*(1, 34) = 0.4750, *p* = 0.4954].

### A global α7 nAChR KO impairs pattern separation in male mice only

**Figure 5:**
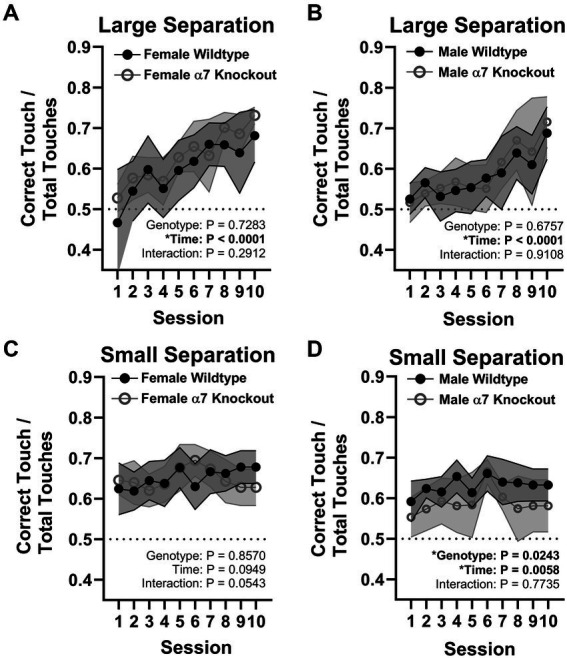
Pattern separation touchscreen task. **(A)** Large separation task for female mice. **(B)** Large separation task for male mice. **(C)** Small separation task for female mice. **(D)** Small separation task for male mice. Data in line graphs are represented by mean ± 95% CI. ^*^represents a significant effect at *p* < 0.05 for that effect.

To determine if the global KO of α7 nAChRs impairs hippocampal function, we utilized a touchscreen task that was generated to mimic the Cambridge Neuropsychological Test Automated Battery (CANTAB), a highly selective test for dementia in humans (Sahgal et al., 1991; Égerházi et al., 2007). Both tests require the subject to touch visual cues in a pattern which precisely tests spatial memory combined with immediate reward. In mice, the small pattern separation task, and not the large, has been found to require juvenile and adult neurogenesis within the dentate gyrus (Clelland et al., 2009). α7 nAChR KO and WT mice were tested on large and small pattern separation tasks at 4 months old. During the training phase, 50% of mice reached competence (at least 85% correct touches) by the fourth session, 82% by the fifth session, 95% by the sixth session, and 100% by the seventhe. As expected, there were no effects of genotype in the large separation task for females [*F*(1, 16) = 1.192, *p* = 0.2912; Figure 5A] or males [*F*(1, 18) = 0.1809, *p* = 0.6757; Figure 5B]. There were no effects of genotype in the small separation task for female mice [*F*(1, 16) = 0.03355, *p* = 0.8570; Figure 5D]. However, KO male mice performed on average 7% worse than WT male mice in the small separation task [*F*(1, 18) = 6.047, *p* = 0.0243; Figure 5C], indicating a male-specific behavioral impairment.

To rule out differences in motivation, we calculated the average reward latency (how long it takes the mice to retrieve the reward after making a correct choice) and the average selection latency (how long it takes to make a response at the beginning of a trial) across trials for the small separation task. We found no differences in reward latency [Genotype: *F*(1, 34) = 0.0009959, *p* = 0.9750, Sex: *F*(1, 34) = 1.408, *p* = 0.2436; Interaction: *F*(1, 34) = 0.4567, *p* = 0.5037] or choice latency [Genotype: *F*(1, 34) = 0.8712, *p* = 0.3572, Sex: *F*(1, 34) = 0.5026, *p* = 0.4832; Interaction: *F*(1, 34) = 0.8008, *p* = 0.3771] indicating that motivational differences cannot explain the sex-dependent deficit in the KO mice.

### Analyses of behavioral strategies

To explore differences in performance strategies and to gain insight into the lack of a pattern separation deficit in female KOs, we performed *ad hoc* analyses on the strategy of groups using raw trial data from the small separation trials with genotype and sex as main factors. We focused on three different approaches: (1) comparing performance before and after the first reversal in order to analyze whether the performance differs between the initial reversal contingency and subsequent performance (Swan et al., 2014), (2) fitting a GLM on the behavioral strategy to determine side bias, tendency to stay or shift sides, and a tendency to stay or shift sides based on the outcome of the previous choice (Chen et al., 2021), and (3) categorizing the primary strategies used by each group.

**Figure 6:**
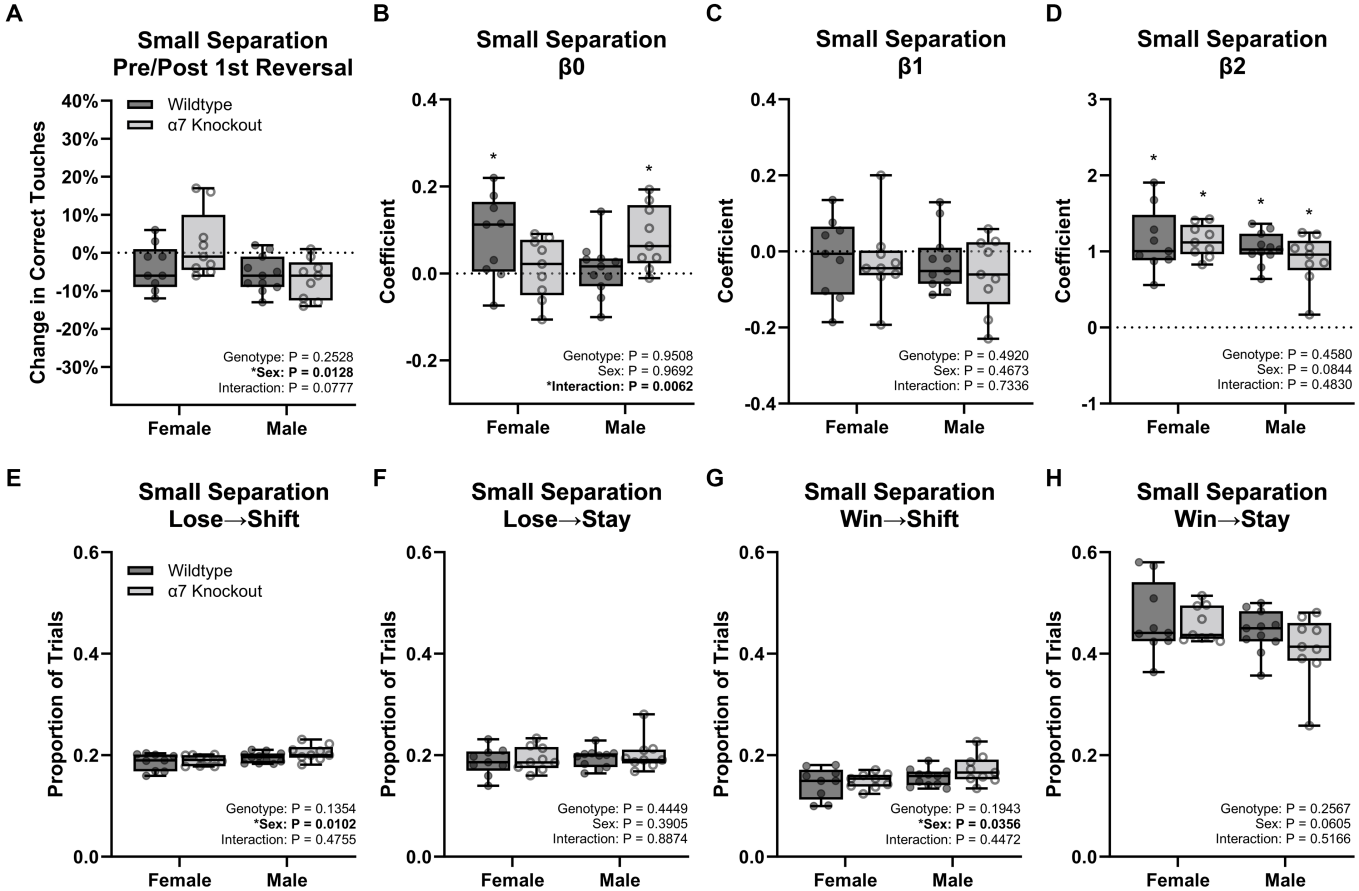
Behavior strategy for the small separation touchscreen task. **(A)** Percent change in correct touches before and after the first reversal. **(B)** Estimates of β_0_. **(C)** Estimates of β_1_. **(D)** Estimates of β_2_. **(E)** Proportion of trials where mice shifted sides after an incorrect choice. **(F)** Proportion of trials where mice stayed on the same side after an incorrect choice. **(G)** Proportion of trials where mice shifted sides after a correct choice. **(H)** Proportion of trials where mice stayed on the same side after a correct choice. Boxplots represent median, interquartile range, absolute range, and individual points for each animal. ^*^represents a significant effect at *p* < 0.05 for the related effect or a specific group versus zero for panels **(B,D)**.

Successful performance of this task requires the animal to alternate their response to the right or left based on the current contingency. In each session the left side was always designated as initially correct. To determine how this initial contingency shift affects subsequent performance, we analyzed percent correct during the initial reversal contingency versus all subsequent contingencies. This analysis (Figure 6A) showed males performed worse after the first reversal compared to the females [*F*(1, 34) = 6.903, *p* = 0.0128] with no effect of genotype [*F*(1, 34) = 1.353, *p* = 0.2528], and no interaction [*F*(1, 34) = 3.309, *p* = 0.0777]. This intriguing finding argues that males are using a unique behavioral strategy affected by the initial reversal contingency, which may be exacerbated by the KO.

We next modeled the strategies using a GLM with three components: β_0_ to represent an overall side bias (left versus right), β_1_ to represent the tendency to stay or switch sides regardless of the previous reward outcome, and β_2_ to represent the tendency to stay or switch sides based on the previous reward outcome (Supplementary Figures 1–4). The β_0_ coefficients (Figure 6B) were small, but significantly greater than zero for WT females (*p* = 0.0333) and knockout males (*p* = 0.0093) indicating a slight left-side bias for these groups. This small effect is likely due to the first trial always being on the left side. Between groups, there was no effect of genotype [*F*(1, 34) = 0.003864, *p* = 0.9508] or sex [*F*(1, 34) = 0.001513, *p* = 0.9692], but a significant interaction was present [*F*(1, 34) = 8.514, *p* = 0.0062]. However, no Tukey post-hoc comparison was significant for the interaction. The β_1_ coefficient (Figure 6C) was not significantly different than zero for any group and did not have a significant effect of genotype [*F*(1, 34) = 0.5405, *p* = 0.4673], sex [*F*(1, 34) = 0.4826, *p* = 0.4920], or an interaction [*F*(1, 34) = 0.1178, *p* = 0.7336] indicating no group had an overall tendency to shift or stay regardless of the previous trial. Analysis of the β_2_ coefficient (Figure 6D) was significantly greater than zero for each group (*p* < 0.0001 for each comparison) indicating a dominant strategy to stay when previously correct, and to shift when previously incorrect. Between groups, there was no significant effect of genotype [*F*(1, 34) = 0.5405, *p* = 0.4673], sex [*F*(1, 34) = 0.4826, *p* = 0.4920], or an interaction [*F*(1, 34) = 0.1178, *p* = 0.7336].

We next conducted a trial-by-trial analysis on the small separation task to categorize each trial as Win→Stay, Win→Shift, Lose→Stay, or Lose→Shift, based on the current trial’s choice and previous trial’s outcome (Figures 6E–H). The number of trials for each strategy was then divided by the total trials to determine a proportion. Matching the GLM results, Win→Stay was the dominant strategy (44.5%) followed by Lose→Shift and Lose→Stay (both at 19.4%) and finally Win→Shift (15.6%). Males were 1.2% more likely to Lose→Shift [*F*(1, 34) = 7.401, *p* = 0.0102] and 1.7% more likely to Win→Shift [*F*(1, 34) = 4.788, *p* = 0.0356] than females. There were no additional effects of genotype, sex, or interactions for any other comparison.

Together, the additional three analysis methods indicate males were more biased by the initial reversal contingency relative to females. While the categorization of trial strategies indicates males had a slightly greater tendency to shift sides regardless of the previous trial’s outcome, the GLM results suggest no difference. In conjunction, this phenomenon is unlikely to be an important contributing factor to differences in performance between sexes. Overall, the difficulty performing the first reversal is the strongest lead in understanding the specific behavioral deficit from the global α7 nAChR KO in males.

## Discussion

In the current study, a global knockout of α7 nAChRs reduced granule and pyramidal cell counts within the dentate gyrus and CA1-3 regions, respectively. Despite both sexes displaying this reduction, only males presented a behavioral deficit in a spatial discrimination/reversal operant touchscreen task. Consistent with our hypothesis, this deficit was specific to the “small separation” version of the task, which has been shown to be dependent on adult neurogenesis (Clelland et al., 2009; Swan et al., 2014).

The knockout of α7 nAChRs may disrupt typical growth and integration of newborn granule via multiple mechanisms such as changes in plasticity (Gu et al., 2012), GABA production through PKA phosphorylation (Bates et al., 2014), inflammation (Campbell et al., 2010), or levels of IGF-1 (Kita et al., 2014). The timing and method of knockout appears to be essential as current and previous findings have reported increased or decreased markers of neurogenesis and related memory dysfunction between sexes (Otto and Yakel, 2019; Nacer et al., 2021). The current results fill a needed conceptual gap by quantifying the total number of primary cells (pyramidal and granule cells) throughout the hippocampus instead of relying on markers of neurogenesis. To our surprise, both primary cells were reduced in equal magnitude indicating the effects of α7 nAChRs likely extend to natal and juvenile growth of both cell types, not just granule cell adult neurogenesis in the dentate gyrus. This finding is important for future studies delineating the specific effects on this circuit and is particularly novel as pyramidal cell formation and/or survival have not previously been connected to α7 nAChRs. Another intriguing finding is the unchanged hippocampal volume despite reductions in granule and pyramidal cells. What structures replaced this lost space, such as other neuron types, increased arborization of existing primary cells, or neuropil, is currently unknown. Although, granule and pyramidal cell layers are relatively thin compared to the entire hippocampus, so *a* ≈ 20% reduction in these cells may not represent a detectable reduction in total hippocampal volume. Still, the impact of α7 nAChRs on overall hippocampal structural integrity is much more profound than previously known. Future work will be required to delineate whether this is primarily a result of differences in neurogenesis, dendritic development, pruning, and/or cell loss.

The deficit in performance in the small separation task is consistent with prior studies that have utilized targeted ablation of adult hippocampal neurogenesis (Clelland et al., 2009; Swan et al., 2014) and the male-specific deficit confirms prior findings in our lab that α7 nAChRs play a sex-dependent role in behavior (Otto and Yakel, 2019; Nacer et al., 2021). It should be noted that while we have previously found the α7 nAChR knockout to alter hippocampal neurogenesis, this phenomenon was not directly investigated in the current study. The lack of a deficit in females, despite the same reduction in pyramidal and granule cell counts, led us to investigate whether sex differences in the underlying strategy could be playing a role (Chen et al., 2021). However, it is possible neurogenesis was not specifically affected by this task in the current model, explaining the lack of a profound deficit. Prior work has argued that adult neurogenesis-mediated deficits in this task may be driven primarily by impaired ability to switch between reversal contingencies, suggestive of a primary deficit in cognitive flexibility rather than pattern separation: e.g., deficits were not observed during the initial reversal contingency but were primarily observed during the subsequent reversals (Swan et al., 2014). Here, we observed a reduction in task performance after the initial reversal contingency in males only regardless of genotype, which argues for an underlying sex difference in cognitive flexibility that could interact with loss of α7 nAChRs (Chen et al., 2021).

Model fitting the behavioral strategy and categorizing the percentage of empirical strategy use showed that the overall dominant strategy across mice was Win→Stay, which combined with Lose→Shift, results in a highly optimal strategy for this task. Based on the trial strategy categorization, males showed a slightly stronger tendency to shift sides regardless of the prior outcome relative to females, which may also contribute to the male-specific deficit. However, the effect was small and the GLM results indicate no differences in Stay/Shift likelihoods. As this in-depth strategy analysis was exploratory, future work will be required to confirm these findings. The touchscreen task employed here was based on prior literature focused on hippocampal neurogenesis-dependent deficits and was therefore not optimized to provide details on behavioral strategy as has been done in other studies (Chen et al., 2021).

Dysfunction associated with the gene encoding α7 nAChRs, CHRNA7, is linked to multiple psychiatric disorders that could be relevant to the current findings, including schizophrenia, bipolar disorder, ADHD, and Alzheimer’s disease (Dineley et al., 2015). In humans, microdeletions within the locus containing CHRNA7 (Masurel-Paulet et al., 2010) and mutations within the partial duplication of the CHRNA7 gene, CHRFAM7A (Sinkus et al., 2015), are the most thoroughly documented of such dysfunctions. However, the consequences of these mutations may not be directly translatable to the current model. While the α7 nAChR knockout approach is specific and effective, compensatory mechanisms may be preventing major deficits to relevant pattern separation circuits. For example, α7 nAChR knockout mice have mixed reports of minimal psychiatric abnormalities (Paylor et al., 1998; Yin et al., 2017), but also reductions of memory function (Hoyle et al., 2006; Young et al., 2007, 2011; Clelland et al., 2009; Otto and Yakel, 2019; Nacer et al., 2021). Additionally, the global knockout leaves the possibility other brain regions or systems were affected by the knockout and could be the source of our results. Despite these limitations, our knockout model underscores the important role of α7 nAChR dysfunction in cognition, adding valuable insights into the molecular underpinnings of cognitive impairments.

In conclusion, our findings show that α7 nAChRs are critical for the proper formation of the hippocampus and play a sex-dependent role in cognitive behavioral performance. We hypothesized that only granule cells of the dentate gyrus would be affected based on prior studies, however, the unexpected discovery of the loss of pyramidal cells in CA1-3 highlights the need for further investigation into the role of α7 nAChRs in brain development and its implications. The male-specific deficit in performance in a spatial pattern separation task is also consistent with prior findings. Our in-depth analysis of the behavioral strategies provides some insights to potentially explain this sex difference, however further work is needed to fully understand the behavioral resilience in females. This sex-specific impairment could provide insight into the sex differences in the human population for disorders that have been associated with α7 nAChRs (Arnett et al., 2015; Mendrek and Mancini-Marïe, 2016; Ferretti et al., 2018; Reddy et al., 2021; Menculini et al., 2022).

## Data availability statement

The original contributions presented in the study are included in the article/Supplementary material, further inquiries can be directed to the corresponding authors.

## Ethics statement

The animal study was approved by NIEHS/NIH Humane Care and Use of Animals Protocols. The study was conducted in accordance with the local legislation and institutional requirements.

## Author contributions

AL, KS, JC, and JY designed research. AL, KS, SN, and JD performed research. AL, GL, and JC analyzed data. AL wrote the manuscript. SN, KS, GL, JD, JC, and JY provided edits. All authors contributed to the article and approved the submitted version.

## Funding

This work was supported by the Intramural Research Program of the National Institute of Environmental Health Sciences, National Institutes of Health (Z1ES90998) and contract GS-00F-173CA-75N96021F00109 to Social & Scientific Systems Inc., a DLH Holdings Corp. company. The funder was not involved in the study design, collection, analysis, interpretation of data, the writing of this article, or the decision to submit it for publication.

## Conflict of interest

GL was employed by Social & Scientific Systems, Inc., a DLH Holdings Corp. Company, and received funding from contract GS-00F-173CA-75N96021F00109 to assist in analyzing behavioral data.

The remaining authors declare that the research was conducted in the absence of any commercial or financial relationships that could be construed as a potential conflict of interest.

## Publisher’s note

All claims expressed in this article are solely those of the authors and do not necessarily represent those of their affiliated organizations, or those of the publisher, the editors and the reviewers. Any product that may be evaluated in this article, or claim that may be made by its manufacturer, is not guaranteed or endorsed by the publisher.

## Supplementary material

The Supplementary material for this article can be found online at: https://www.frontiersin.org/articles/10.3389/fnins.2023.1244118/full#supplementary-material

SUPPLEMENTARY FIGURE S1 Session details of the three component General Linear Model for analysis of behavioral strategy in Wildtype Male mice. Left, in red: average model fit values across all mice for the large separation task. Right, in blue, average model fit values across all mice for the small separation task. Error bars are +/- Standard Error.

SUPPLEMENTARY FIGURE S2 Session details of the three component General Linear Model for analysis of behavioral strategy in Wildtype Female mice. Left, in red: average model fit values across all mice for the large separation task. Right, in blue, average model fit values across all mice for the small separation task. Error bars are +/- Standard Error.

SUPPLEMENTARY FIGURE S3 Session details of the three component General Linear Model for analysis of behavioral strategy in A7 Knockout Male mice. Left, in red: average model fit values across all mice for the large separation task. Right, in blue, average model fit values across all mice for the small separation task. Error bars are +/- Standard Error.

SUPPLEMENTARY FIGURE S4 Session details of the three component General Linear Model for analysis of behavioral strategy in A7 Knockout Female mice. Left, in red: average model fit values across all mice for the large separation task. Right, in blue, average model fit values across all mice for the small separation task. Error bars are +/- Standard Error.

SUPPLEMENTARY TABLE S1 Details of the statistical analyses separated by tabs.

SUPPLEMENTARY VIDEO S1 Example video of a well-trained mouse (wild type female) performing the small separation task.

## Footnotes

^1^ https://www.jax.org/strain/003232

^2^ https://www.jax.org/strain/000664

## References

[ref1] Arnett A. B. Pennington B. F. Willcutt E. G. DeFries J. C. Olson R. K. (2015). Sex differences in ADHD symptom severity. J. Child Psychol. Psychiatry 56, 632–639. doi: 10.1111/jcpp.12337, PMID: 25283790 PMC4385512

[ref2] Bates R. C. Stith B. J. Stevens K. E. Adams C. E. (2014). Reduced Chrna7 expression in C3H mice is associated with increases in hippocampal parvalbumin and glutamate decarboxylase-67 (GAD67) as well as altered levels of GABAA receptor subunits. Neuroscience 273, 52–64. doi: 10.1016/j.neuroscience.2014.05.004, PMID: 24836856 PMC4122271

[ref3] Briley D. Ghirardi V. Woltjer R. Renck A. Zolochevska O. Taglialatela G.. (2016). Preserved neurogenesis in non-demented individuals with AD neuropathology. Sci. Rep. 6:27812. doi: 10.1038/srep27812 27298190 PMC4906289

[ref4] Campbell N. R. Fernandes C. C. Halff A. W. Berg D. K. (2010). Endogenous signaling through α7-containing nicotinic receptors promotes maturation and integration of adult-born neurons in the hippocampus. J. Neurosci. 30, 8734–8744. doi: 10.1523/JNEUROSCI.0931-10.2010, PMID: 20592195 PMC2905643

[ref5] Chacón M. A. Reyes A. E. Inestrosa N. C. (2003). Acetylcholinesterase induces neuronal cell loss, astrocyte hypertrophy and behavioral deficits in mammalian hippocampus. J. Neurochem. 87, 195–204. doi: 10.1046/j.1471-4159.2003.01985.x, PMID: 12969266

[ref6] Chen C. S. Ebitz R. B. Bindas S. R. Redish A. D. Hayden B. Y. Grissom N. M. (2021). Divergent strategies for learning in males and females. Curr. Biol. 31, 39–50.e4. doi: 10.1016/j.cub.2020.09.075, PMID: 33125868 PMC8120733

[ref7] Clelland C. D. Choi M. Romberg C. Clemenson G. D. Fragniere A. Tyers P.. (2009). A functional role for adult hippocampal neurogenesis in spatial pattern separation. Science 325, 210–213. doi: 10.1126/science.1173215, PMID: 19590004 PMC2997634

[ref8] Conejero-Goldberg C. Davies P. Ulloa L. (2008). Alpha7 nicotinic acetylcholine receptor: a link between inflammation and neurodegeneration. Neurosci. Biobehav. Rev. 32, 693–706. doi: 10.1016/j.neubiorev.2007.10.007, PMID: 18180036 PMC2895566

[ref9] Davies P. Maloney A. J. (1976). Selective loss of central cholinergic neurons in Alzheimer’s disease. Lancet 2:1403. doi: 10.1016/s0140-6736(76)91936-x, PMID: 63862

[ref10] Delotterie D. F. Mathis C. Cassel J. C. Rosenbrock H. Dorner-Ciossek C. Marti A. (2015). Touchscreen tasks in mice to demonstrate differences between hippocampal and striatal functions. Neurobiol. Learn. Mem. 120, 16–27. doi: 10.1016/j.nlm.2015.02.007, PMID: 25687692

[ref11] Dineley K. T. Pandya A. A. Yakel J. L. (2015). Nicotinic ACh receptors as therapeutic targets in CNS disorders. Trends Pharmacol. Sci. 36, 96–108. doi: 10.1016/j.tips.2014.12.002, PMID: 25639674 PMC4324614

[ref12] Égerházi A. Berecz R. Bartók E. Degrell I. (2007). Automated neuropsychological test battery (CANTAB) in mild cognitive impairment and in Alzheimer’s disease. Prog. Neuro-Psychopharmacol. Biol. Psychiatry 31, 746–751. doi: 10.1016/j.pnpbp.2007.01.011 17289240

[ref13] Ferretti M. T. Iulita M. F. Cavedo E. Chiesa P. A. Schumacher Dimech A. Santuccione Chadha A.. (2018). Sex differences in Alzheimer disease—the gateway to precision medicine. Nat. Rev. Neurol. 14, 457–469. doi: 10.1038/s41582-018-0032-9, PMID: 29985474

[ref14] Fontana I. C. Kumar A. Nordberg A. (2023). The role of astrocytic α7 nicotinic acetylcholine receptors in Alzheimer disease. Nat. Rev. Neurol. 19, 278–288. doi: 10.1038/s41582-023-00792-4, PMID: 36977843

[ref15] Gardi J. E. Nyengaard J. R. Gundersen H. J. G. (2008). The proportionator: unbiased stereological estimation using biased automatic image analysis and non-uniform probability proportional to size sampling. Comput. Biol. Med. 38, 313–328. doi: 10.1016/j.compbiomed.2007.11.002, PMID: 18163985

[ref16] Gu Z. Lamb P. W. Yakel J. L. (2012). Cholinergic coordination of presynaptic and postsynaptic activity induces timing-dependent hippocampal synaptic plasticity. J. Neurosci. 32, 12337–12348. doi: 10.1523/JNEUROSCI.2129-12.2012, PMID: 22956824 PMC3474164

[ref17] Gu Z. Yakel J. L. (2011). Timing-dependent septal cholinergic induction of dynamic hippocampal synaptic plasticity. Neuron 71, 155–165. doi: 10.1016/j.neuron.2011.04.026, PMID: 21745645 PMC3134790

[ref18] Gundersen H. J. G. Jensen E. B. (1987). The efficiency of systematic sampling in stereology and its prediction*. J. Microsc. 147, 229–263. doi: 10.1111/j.1365-2818.1987.tb02837.x, PMID: 3430576

[ref19] Gundersen H. J. G. Mirabile R. Brown D. Boyce R. W. (2013). “Chapter 8—stereological principles and sampling procedures for Toxicologic pathologists” in Haschek and Rousseaux’s Handbook of Toxicologic Pathology. eds. Haschek W. M. Rousseaux C. G. Wallig M. A.. 3rd Edn. (London, UK: Academic Press), 215–286.

[ref01] Howard C. V. Reed M. G. (2005). Unbiased Stereology: Three-Dimensional Measurement in Microscopy. 2nd Edn. Oxford: Bios Scientific Publishers.

[ref20] Hoyle E. Genn R. F. Fernandes C. Stolerman I. P. (2006). Impaired performance of alpha7 nicotinic receptor knockout mice in the five-choice serial reaction time task. Psychopharmacology 189, 211–223. doi: 10.1007/s00213-006-0549-2, PMID: 17019565 PMC1705494

[ref21] Jortner B. S. (2006). The return of the dark neuron. A histological artifact complicating contemporary neurotoxicologic evaluation. Neurotoxicology 27, 628–634. doi: 10.1016/j.neuro.2006.03.002, PMID: 16650476

[ref22] Kita Y. Ago Y. Higashino K. Asada K. Takano E. Takuma K.. (2014). Galantamine promotes adult hippocampal neurogenesis via M1 muscarinic and α7 nicotinic receptors in mice. Int. J. Neuropsychopharmacol. 17, 1957–1968. doi: 10.1017/S1461145714000613, PMID: 24818616

[ref23] Letsinger A. C. Gu Z. Yakel J. L. (2022). α7 nicotinic acetylcholine receptors in the hippocampal circuit: taming complexity. Trends Neurosci. 45, 145–157. doi: 10.1016/j.tins.2021.11.006, PMID: 34916082 PMC8914277

[ref24] Løkkegaard A. (2004). “The number of microvessels estimated by an unbiased stereological method applied in a brain region” in Quantitative Methods in Neuroscience. eds. S. M. Evans, A. M. Janson and J. R. Nyengaard (Oxford: Oxford University Press), 167–182.

[ref25] Masurel-Paulet A. Andrieux J. Callier P. Cuisset J. Le Caignec C. Holder M.. (2010). Delineation of 15q13.3 microdeletions. Clin. Genet. 78, 149–161. doi: 10.1111/j.1399-0004.2010.01374.x, PMID: 20236110

[ref26] McTighe S. M. Mar A. C. Romberg C. Bussey T. J. Saksida L. M. (2009). A new touchscreen test of pattern separation: effect of hippocampal lesions. Neuroreport 20, 881–885. doi: 10.1097/WNR.0b013e32832c5eb2, PMID: 19421077

[ref27] Menculini G. Steardo L. Sciarma T. D’Angelo M. Lanza L. Cinesi G.. (2022). Sex differences in bipolar disorders: impact on psychopathological features and treatment response. Front. Psychiatry 13:926594. doi: 10.3389/fpsyt.2022.926594, PMID: 35757228 PMC9226371

[ref28] Mendrek A. Mancini-Marïe A. (2016). Sex/gender differences in the brain and cognition in schizophrenia. Neurosci. Biobehav. Rev. 67, 57–78. doi: 10.1016/j.neubiorev.2015.10.013, PMID: 26743859

[ref29] Mohammad D. Chan P. Bradley J. Lanctôt K. Herrmann N. (2017). Acetylcholinesterase inhibitors for treating dementia symptoms—a safety evaluation. Expert Opin. Drug Saf. 16, 1009–1019. doi: 10.1080/14740338.2017.1351540 28678552

[ref30] Moreno-Jiménez E. P. Flor-García M. Terreros-Roncal J. Rábano A. Cafini F. Pallas-Bazarra N.. (2019). Adult hippocampal neurogenesis is abundant in neurologically healthy subjects and drops sharply in patients with Alzheimer’s disease. Nat. Med. 25, 554–560. doi: 10.1038/s41591-019-0375-9, PMID: 30911133

[ref31] Nacer S. A. Letsinger A. C. Otto S. Strauss J. Viktoriya D. Riddick N. V.. (2021). Loss of α7 nicotinic acetylcholine receptors in GABAergic neurons causes sex—dependent decreases in radial glia—like cell quantity and impairments in cognitive and social behavior. Brain Struct. Funct. 226, 365–379. doi: 10.1007/s00429-020-02179-3, PMID: 33398432 PMC8121181

[ref32] Nakashiba T. Cushman J. D. Pelkey K. A. Renaudineau S. Buhl D. L. McHugh T. J.. (2012). Young dentate granule cells mediate pattern separation, whereas old granule cells facilitate pattern completion. Cells 149, 188–201. doi: 10.1016/j.cell.2012.01.046, PMID: 22365813 PMC3319279

[ref33] Newhouse P. (2004). Effects of nicotinic stimulation on cognitive performance. Curr. Opin. Pharmacol. 4, 36–46. doi: 10.1016/j.coph.2003.11.001 15018837

[ref34] Orr-Urtreger A. Göldner F. M. Saeki M. Lorenzo I. Goldberg L. De Biasi M.. (1997). Mice deficient in the α7 neuronal nicotinic acetylcholine receptor lack α-Bungarotoxin binding sites and hippocampal fast nicotinic currents. J. Neurosci. 17, 9165–9171. doi: 10.1523/JNEUROSCI.17-23-09165.1997, PMID: 9364063 PMC6573618

[ref35] Otto S. L. Yakel J. L. (2019). The α7 nicotinic acetylcholine receptors regulate hippocampal adult-neurogenesis in a sexually dimorphic fashion. Brain Struct. Funct. 224, 829–846. doi: 10.1007/s00429-018-1799-6, PMID: 30515567 PMC6432768

[ref36] Paylor R. Nguyen M. Crawley J. N. Patrick J. Beaudet A. Orr-Urtreger A. (1998). α7 nicotinic receptor subunits are not necessary for hippocampal-dependent learning or sensorimotor gating: a behavioral characterization of Acra7-deficient mice. Learn. Mem. 5, 302–316. doi: 10.1101/lm.5.4.302 10454356 PMC311270

[ref37] Reddy D. S. Thompson W. Calderara G. (2021). Molecular mechanisms of sex differences in epilepsy and seizure susceptibility in chemical, genetic and acquired epileptogenesis. Neurosci. Lett. 750:135753. doi: 10.1016/j.neulet.2021.135753, PMID: 33610673 PMC7994197

[ref38] Sahgal A. Sahakian B. J. Robbins T. W. Wray C. J. Lloyd S. Cook J. H.. (1991). Detection of visual memory and learning deficits in Alzheimer’s disease using the Cambridge neuropsychological test automated battery. Dement. Geriatr. Cogn. Disord. 2, 150–158. doi: 10.1159/000107190

[ref39] Schliebs R. Arendt T. (2011). The cholinergic system in aging and neuronal degeneration. Behav. Brain Res. 221, 555–563. doi: 10.1016/j.bbr.2010.11.058 21145918

[ref40] Sinkus M. L. Graw S. Freedman R. Ross R. G. Lester H. A. Leonard S. (2015). The human CHRNA7 and CHRFAM7A genes: a review of the genetics, regulation, and function. Neuropharmacology 96, 274–288. doi: 10.1016/j.neuropharm.2015.02.006, PMID: 25701707 PMC4486515

[ref41] Swan A. A. Clutton J. E. Chary P. K. Cook S. G. Liu G. G. Drew M. R. (2014). Characterization of the role of adult neurogenesis in touch-screen discrimination learning. Hippocampus 24, 1581–1591. doi: 10.1002/hipo.22337, PMID: 25074617 PMC4236255

[ref42] Talpos J. C. McTighe S. M. Dias R. Saksida L. M. Bussey T. J. (2010). Trial-unique, delayed nonmatching-to-location (TUNL): a novel, highly hippocampus-dependent automated touchscreen test of location memory and pattern separation. Neurobiol. Learn. Mem. 94, 341–352. doi: 10.1016/j.nlm.2010.07.006, PMID: 20692356 PMC2989449

[ref43] Yin J. Chen W. Yang H. Xue M. Schaaf C. P. (2017). Chrna7 deficient mice manifest no consistent neuropsychiatric and behavioral phenotypes. Sci. Rep. 7:39941. doi: 10.1038/srep39941, PMID: 28045139 PMC5206704

[ref44] Young J. W. Crawford N. Kelly J. S. Kerr L. E. Marston H. M. Spratt C.. (2007). Impaired attention is central to the cognitive deficits observed in alpha 7 deficient mice. Eur. Neuropsychopharmacol. 17, 145–155. doi: 10.1016/j.euroneuro.2006.03.008 16650968

[ref45] Young J. W. Meves J. M. Tarantino I. S. Caldwell S. Geyer M. A. (2011). Delayed procedural learning in α7-nicotinic acetylcholine receptor knockout mice. Genes Brain Behav. 10, 720–733. doi: 10.1111/j.1601-183X.2011.00711.x, PMID: 21679297 PMC3918170

[ref46] Zhu H. Yan H. Tang N. Li X. Pang P. Li H.. (2017). Impairments of spatial memory in an Alzheimer’s disease model via degeneration of hippocampal cholinergic synapses. Nat. Commun. 8:1676. doi: 10.1038/s41467-017-01943-0, PMID: 29162816 PMC5698429

[ref47] Zhuo J. M. Tseng H. A. Desai M. Bucklin M. E. Mohammed A. I. Robinson N. T.. (2016). Young adult born neurons enhance hippocampal dependent performance via influences on bilateral networks. elife 5:25. doi: 10.7554/eLife.22429, PMID: 27914197 PMC5156524

