# Supplementary figures and images for: Genetic deletion of α7 nAChRs reduces hippocampal granule and pyramidal cell number in both sexes but impairs pattern separation in males only
Source: Front Neurosci. 2023 Sep 7;17:1244118. doi: 10.3389/fnins.2023.1244118 (PMC10513752; doi:10.3389/fnins.2023.1244118)

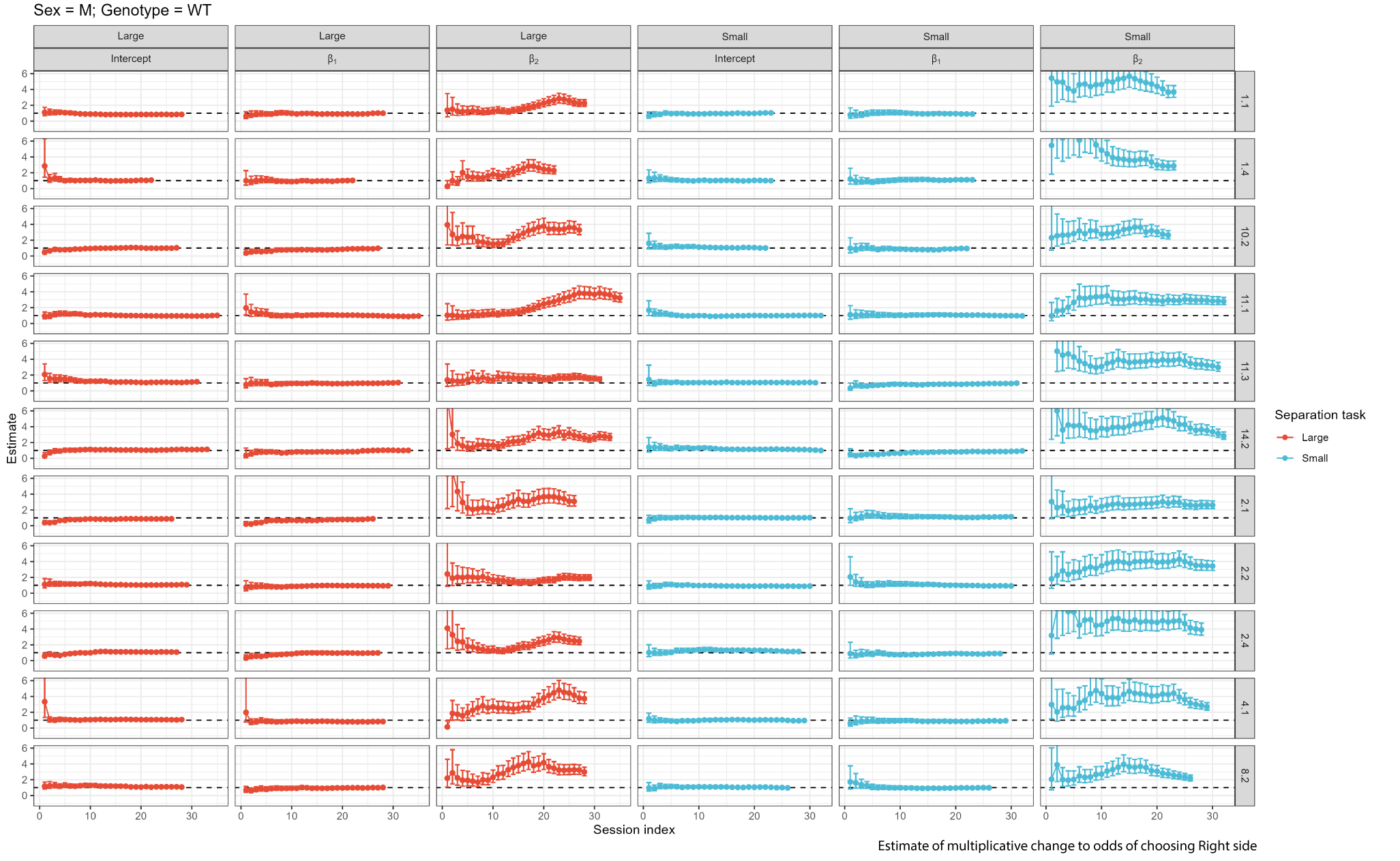

Supplement: SUPPLEMENTARY FIGURE S1 — Session details of the three component General Linear Model for analysis of behavioral strategy in Wildtype Male mice. Left, in red: average model fit values across all mice for the large separation task. Right, in blue, average model fit values across all mice for the small separation task. Error bars are +/- Standard Error. [file Image_1.TIF]

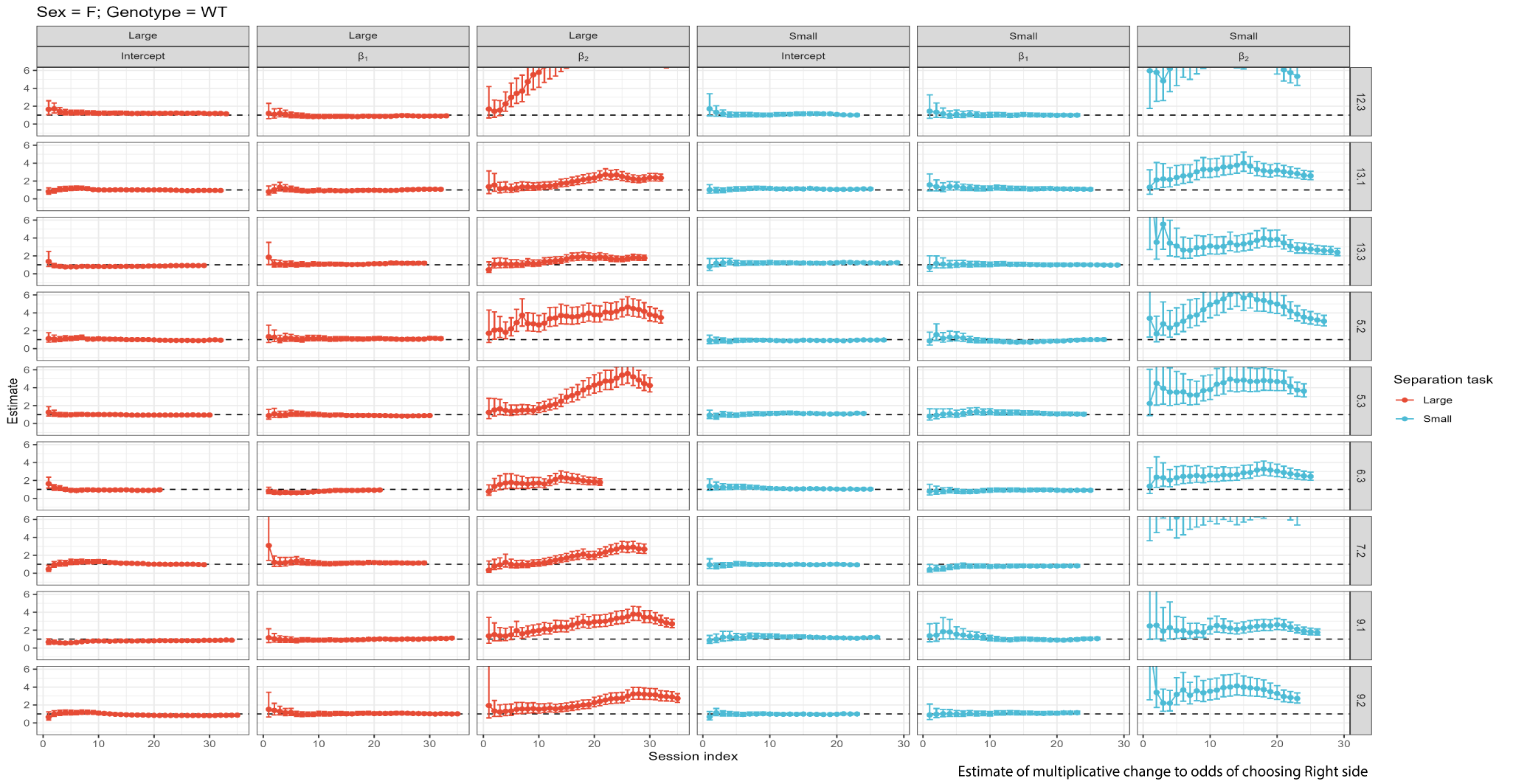

Supplement: SUPPLEMENTARY FIGURE S2 — Session details of the three component General Linear Model for analysis of behavioral strategy in Wildtype Female mice. Left, in red: average model fit values across all mice for the large separation task. Right, in blue, average model fit values across all mice for the small separation task. Error bars are +/- Standard Error. [file Image_2.TIF]

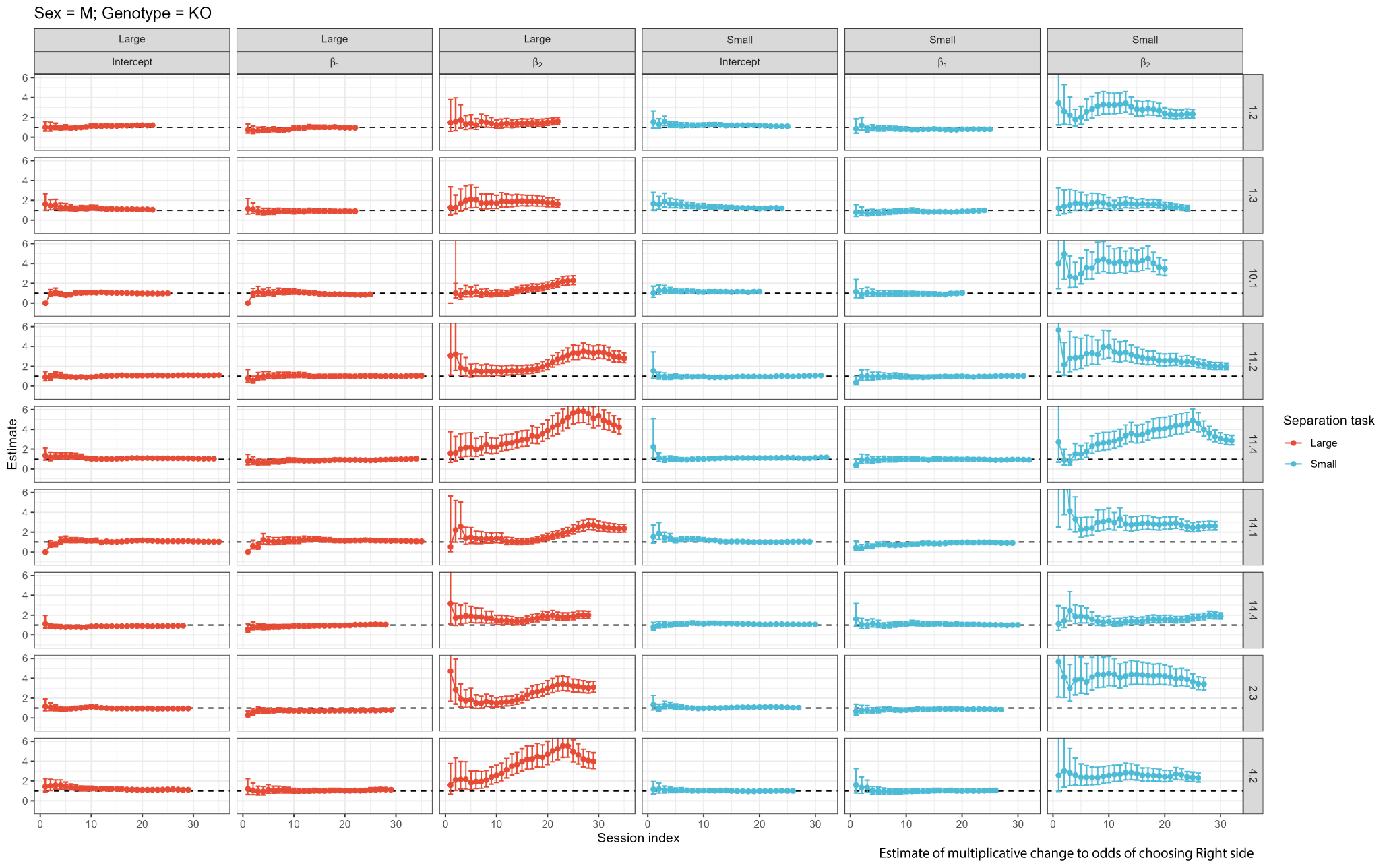

Supplement: SUPPLEMENTARY FIGURE S3 — Session details of the three component General Linear Model for analysis of behavioral strategy in A7 Knockout Male mice. Left, in red: average model fit values across all mice for the large separation task. Right, in blue, average model fit values across all mice for the small separation task. Error bars are +/- Standard Error. [file Image_3.TIF]

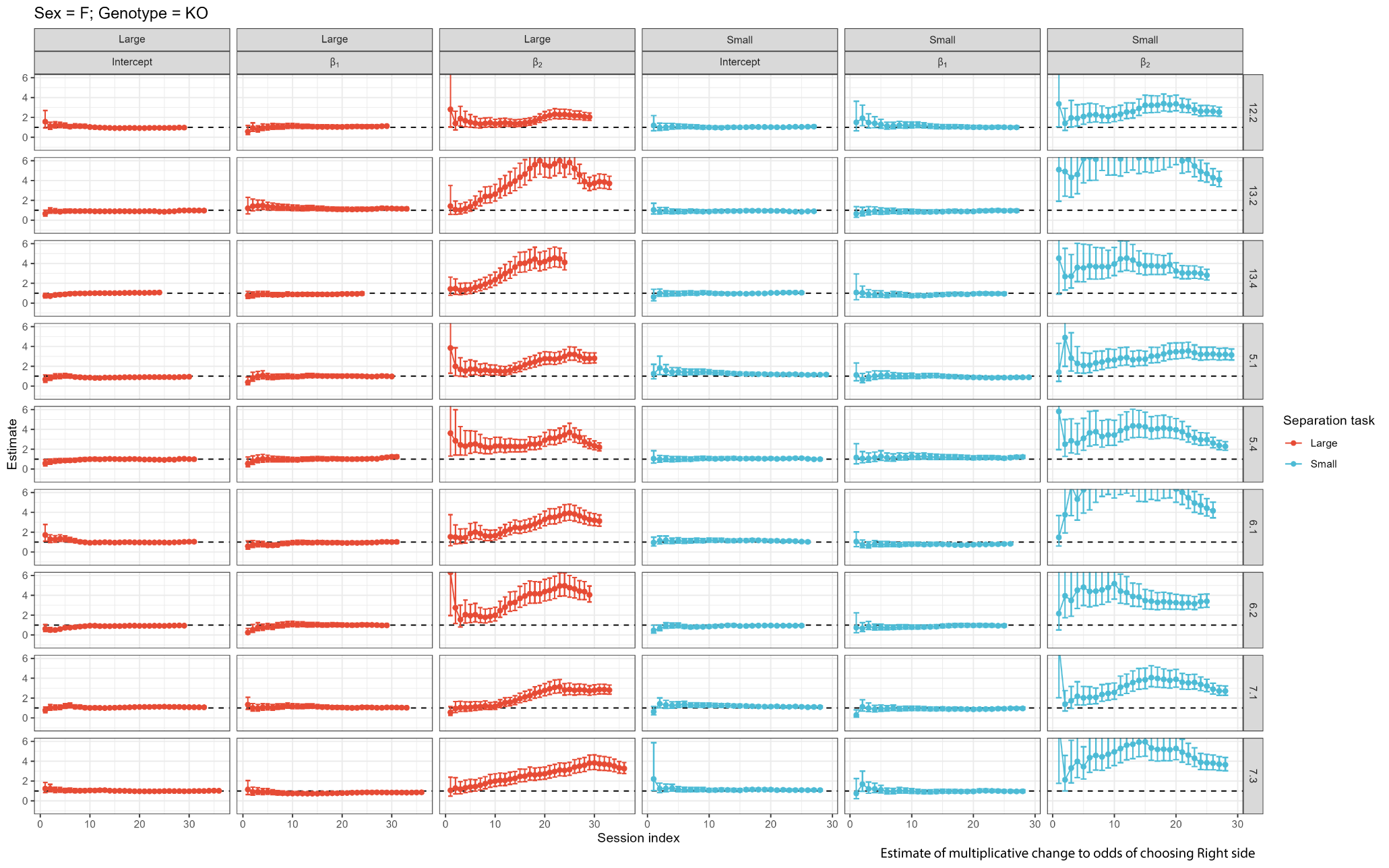

Supplement: SUPPLEMENTARY FIGURE S4 — Session details of the three component General Linear Model for analysis of behavioral strategy in A7 Knockout Female mice. Left, in red: average model fit values across all mice for the large separation task. Right, in blue, average model fit values across all mice for the small separation task. Error bars are +/- Standard Error. [file Image_4.TIF]
